# Antimicrobial resistance of *Salmonella* from poultry meat in Brazil: results of a nationwide survey

**DOI:** 10.1017/S0950268821002156

**Published:** 2021-10-08

**Authors:** Renata Batista Rau, Aldemir Reginato Ribeiro, Amaury dos Santos, Afonso Luís Barth

**Affiliations:** 1 Laboratório Federal de Defesa Agropecuária – RS (LFDA-RS), Ministério da Agricultura, Pecuária e Abastecimento (MAPA) – Estrada da Ponta Grossa, 3036, Porto Alegre, RS, Brazil; 2 Laboratório de Pesquisa em Resistência Bacteriana (LABRESIS), Hospital de Clínicas de Porto Alegre (HCPA) – Rua Ramiro Barcelos, 2350, Porto Alegre, RS, Brazil; 3 Programa de Pós-Graduação em Ciências Farmacêuticas (PPGCF), Universidade Federal do Rio Grande do Sul (UFRGS) – Avenida Ipiranga, 2752, Porto Alegre, RS, Brazil; 4 Laboratório Federal de Defesa Agropecuária – SP (LFDA-SP), Ministério da Agricultura, Pecuária e Abastecimento (MAPA) – Rua Raul Ferrari, s/n, Campinas, SP, Brazil

**Keywords:** Heidelberg, Minnesota, multidrug resistance, One Health, poultry meat, *Salmonella*

## Abstract

The use of antimicrobials in food-producing animals can lead to increased bacterial resistance. Important information to address this problem can be provided by monitoring antimicrobial resistance (AMR) in foodborne pathogens. As part of preliminary activities for the implementation of AMR surveillance in Brazil, a nationwide survey on AMR in *Salmonella enterica* isolates from poultry meat was conducted. The survey evaluated 146 *Salmonella* isolates from poultry meat in 2014, and 163 isolates obtained in 2017. Minimal inhibitory concentrations of 13 antimicrobials were determined by broth microdilution, and isolates were assigned to serotypes by automated ribotyping. High resistance rates were found in 2014 and 2017, in particular to nalidixic acid (84/146, 57.5% and 141/163, 86.5%, respectively), ampicillin (82/146, 56.2% and 125/163, 76.7%), cefotaxime (76/146, 52.1% and 124/163, 76.1%), ceftazidime (73/146, 50.0% and 124/163, 76.1%), ciprofloxacin (83/146, 56.9% and 145/163, 89.0%) and tetracycline (88/146, 60.3% and 135/163, 82.8%). There was a significant increase in resistance to these antibiotics in the second survey period. *Salmonella* ser. Heidelberg and *Salmonella* ser. Minnesota were the main serotypes expressing resistance to these antimicrobials. Multidrug resistance was found in 50.7% (74/146) of the isolates from 2014, and in 77.3% (126/163) of isolates from 2017 (*P* < 0.05). None of the isolates was resistant to azithromycin or meropenem. These findings indicate high and increasing rates of resistance among *Salmonella* from poultry meat in Brazil, mainly associated with *Salmonella ser.* Heidelberg and *Salmonella ser.* Minnesota, stressing the importance of continuous monitoring of AMR in the poultry chain.

## Introduction

Antimicrobial resistance (AMR) is a problem of increasing public health concern worldwide, as antibiotics are among the most prescribed classes of drugs in human medicine. However, they are also largely used for therapy of a wide range of infections in animals and as prophylactic agents to prevent the development of infections in herds, as well as growth promoters in healthy livestock. Such extensive usage has been widely implicated in the selection of bacterial resistance [1]. To combat this effectively, it is recognised that a multidisciplinary approach including human and animal health, food production and environmental factors is necessary, in alignment with the World Health Organization One Health concepts [2].

The indiscriminate use of antibiotics in food-producing animals can lead to bacterial resistance, which can be transmitted to humans through three distinct ways: (i) by direct contact between humans and animals; (ii) through preparation and consumption of contaminated food, and (iii) indirectly, due to the excretion of resistant bacteria and unmetabolised antibiotics by animals, thereby causing additional selective pressure in the environment [3]. Thus, it is very important to include the food production chain in surveillance programmes on AMR, to provide information that enables the adoption of measures of prevention and control of AMR in this sector.

According to the WHO recommendations, integrated surveillance programmes should encompass foodborne pathogens or sentinel microorganisms in human clinical samples, retail foods and healthy food production animals, in this order of priority. It is noteworthy that food of animal origin represents an important route of human exposure to antimicrobial-resistant pathogens. *Salmonella* is usually one of the bacterial groups considered to be of high priority in surveillance programmes in the food chain [4].

A recent WHO report has highlighted that the development and implementation of national action plans on AMR is occurring in different stages among countries [5]. As such plans have recently been developed in Brazil [6, 7], the objective of this study was to provide baseline information to support the construction of a national integrated surveillance programme on AMR in foodborne pathogens in the country. Considering that Brazil is the second largest producer of poultry meat in the world (13 245 million tons) and exports approximately 32% of its production to the international market [8], we have investigated the serotype distribution and AMR of *Salmonella* isolates recovered from this production chain in Brazil, in two different years, 2014 and 2017, to identify possible trends or changes over this period.

## Materials and methods

### Isolates selection

A total of 170 *Salmonella* isolates from each assessed year (2014 and 2017) were randomly selected in a systematic model from the Brazilian Ministry of Agriculture, Livestock and Food Supply (MAPA) collection. The number of isolates examined was based on the Decision 2013/652/EU from the European Commission [9], which proposed that a sample size of 170 isolates per year for antimicrobial susceptibility testing was required for countries producing more than 100 000 tons of slaughtered poultry meat per year. However, due to problems in the storage conditions of the collection, we were only able to recover and test 146 and 163 *Salmonella* isolates from the years of 2014 and 2017, respectively.

### *Salmonella* identification and serotyping

All isolates were obtained from poultry carcasses by the MAPA's laboratory network according to ISO 6579 procedures [10, 11]. Serotypes were determined by automated ribotyping, using RiboPrinter™System (DuPont Qualicon), according to the manufacturer's instructions [12].

### Antimicrobial susceptibility testing

Minimal inhibitory concentrations (MIC) of 13 antimicrobials were determined for the *Salmonella* spp. isolates by broth microdilution, according to ISO 20776-1:2006 [13]. The tested antibiotics are listed in Table 1, as well as the range of concentrations and the EUCAST clinical breakpoints [14]. For those antibiotics without defined breakpoints for *Salmonella*, the results were evaluated according to the European Food Safety Authority (EFSA) recommendations [15]. Isolates were characterised as multidrug-resistant (MDR) if resistant to three or more antimicrobial classes [16]. *Escherichia coli* ATCC 25922, *Staphylococcus aureus* ATCC 29213 and *Pseudomonas aeruginosa* ATCC 27853 were used as quality control strains.

**Table 1.** Antimicrobials range of concentration and interpretative criteria used for testing *Salmonella* isolates from poultry meat

Antimicrobial	Range of concentrations (mg/l)	Interpretative threshold of AMR^a^
Ampicillin (AMP)	0.125–64	>8
Azithromycin (AZM)	0.125–64	>16^b^
Cefotaxime (CTX)	0.01–4	>2
Ceftazidime (CAZ)	0.02–8	>4
Chloramphenicol (CHL)	0.25–128	>8
Ciprofloxacin (CIP)	0.02–8	>0.06
Colistin (COL)	0.03–16	>2
Gentamicin (GEN)	0.06–32	>4
Meropenem (MEM)	0.03–16	>8
Nalidixic acid (NAL)	0.25–128	>16^b^
Tetracycline (TET)	0.125–64	>8^b^
Tigecycline (TGC)	0.02–8	>1^b^
Trimethoprim/sulfamethoxazole (SXT)	0.06–32	>4^c^

a EUCAST clinical breakpoint v 9.0 – European Committee on Antimicrobial Susceptibility Testing resistance breakpoint.

b No current EUCAST clinical breakpoint available. Complementary interpretative thresholds adopted as suggested by EFSA (2019).

c Trimethoprim/sulfamethoxazole in the ratio 1:19. Breakpoints are expressed as the trimethoprim concentration.

### Statistical analysis

Statistical analysis was performed using WinPepi version 11.65. For all proportion estimates, exact binomial 95% confidence intervals (CIs) were calculated. Pearson's *χ*^2^ was used to compare resistance rates between the periods evaluated (*P* < 0.05 was considered statistically significant).

## Results

### Geographical distribution of isolates

**Fig. 1.**
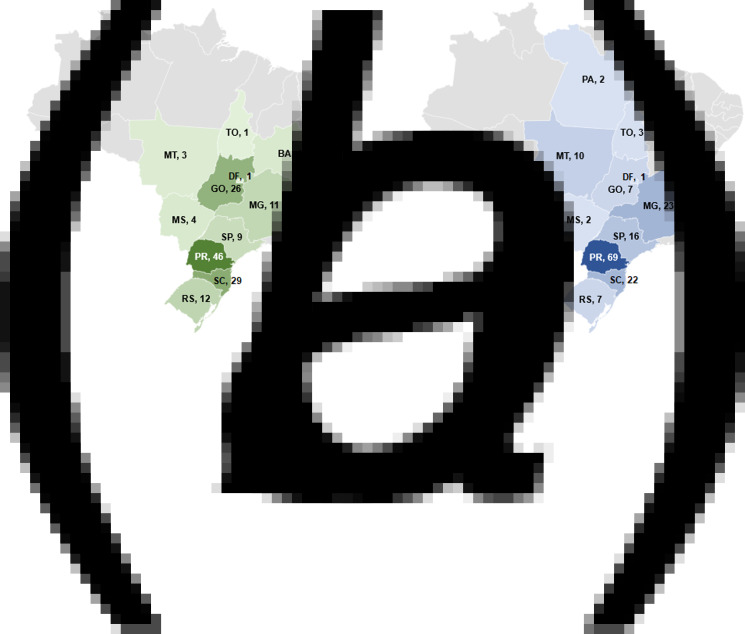
Number of *Salmonella* isolates from 2014 and 2017 selected per state in Brazil. (a) 2014; (b) 2017. BA, Bahia; DF, Distrito Federal; ES, Espírito Santo; GO, Goiás; MG, Minas Gerais; MS, Mato Grosso do Sul; MT, Mato Grosso; PA, Pará; PR, Paraná; RS, Rio Grande do Sul; SC, Santa Catarina; SP, São Paulo; TO, Tocantins.

In the first evaluated period (January to November 2014), the 146 isolates were recovered from 58 different slaughterhouses under federal inspection, in 56 cities and 11 states. In the second period (March to December 2017), the 163 isolates were from 82 slaughterhouses, in 78 cities and 12 states (Fig. 1).

### Serotype distribution

**Fig. 2.**
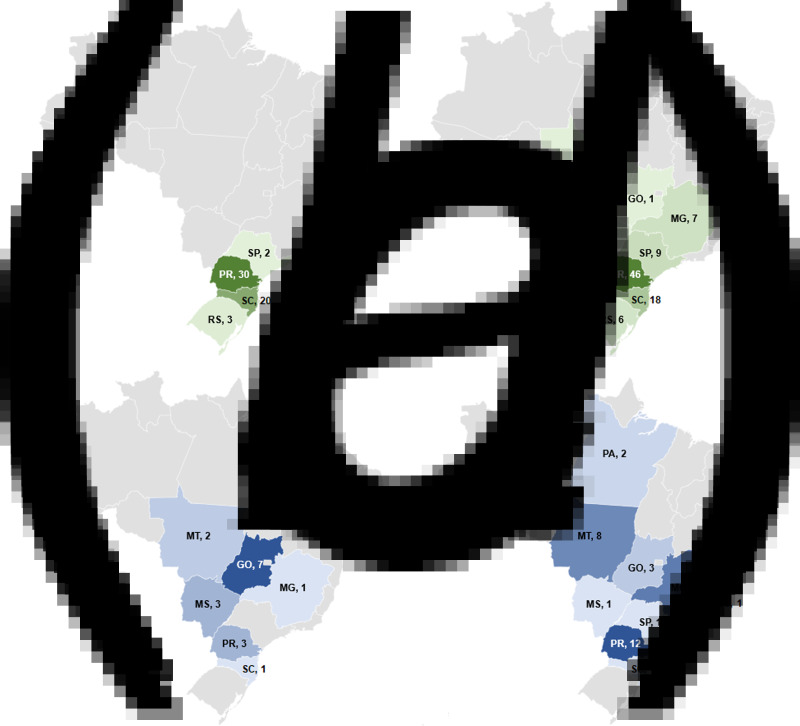
Number of isolates and geographical distribution of the most prevalent serotypes of *Salmonella* from poultry meat in Brazil in 2014 and 2017. (a) Geographic distribution of *Salmonella* ser. Heidelberg in 2014; (b) geographic distribution of *Salmonella* ser. Heidelberg in 2017; (c) geographic distribution of *Salmonella* ser. Minnesota in 2014; (d) geographic distribution of *Salmonella* ser. Minnesota in 2017. ES, Espírito Santo; GO, Goiás; MG, Minas Gerais; MS, Mato Grosso do Sul; MT, Mato Grosso; PA, Pará; PR, Paraná; RS, Rio Grande do Sul; SC, Santa Catarina; SP, São Paulo.

In total, 29 different serotypes were identified among the 146 *Salmonella* isolates from 2014 (Table S1). The most frequent were *Salmonella* ser. Heidelberg (55, 37.7%), *Salmonella* ser. Minnesota (17, 11.6%), *Salmonella* ser. Schwarzengrund (10, 6.8%), *Salmonella* ser. Infantis (9, 6.2%) and *Salmonella* ser. Saintpaul (6, 4.1%). The isolates from 2017 fell into 18 serotypes (Table S1), with *Salmonella* ser. Heidelberg (89, 54.6%), *Salmonella* ser. Minnesota (38, 23.3%) and *Salmonella* ser. Saintpaul (8, 4.9%) being the most frequent. The evaluation of the geographical distribution of the two most prevalent serotypes indicated that *Salmonella* ser. Heidelberg was identified in samples from four states in 2014, and in eight states in 2017. Likewise, *Salmonella* ser. Minnesota was recovered in six and nine states in 2014 and 2017, respectively (Fig. 2).

### Antimicrobial resistance

**Fig. 3.**
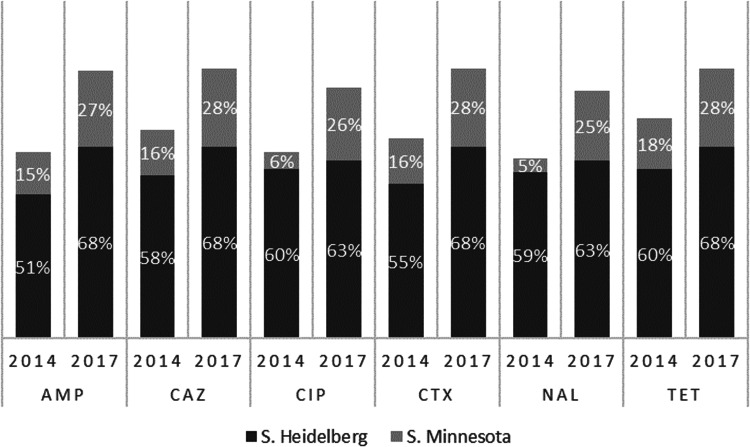
Main resistance rates of the serotypes Heidelberg and Minnesota from poultry meat in Brazil in each year analysed.

Table 2 shows that resistance to nalidixic acid (NAL), ampicillin (AMP), cefotaxime (CTX), ceftazidime (CAZ), ciprofloxacin (CIP) and tetracycline (TET) was the most prevalent in both sampling years and there was a significant increase in resistance to these antibiotics in the most recent survey. Resistance of the two prevalent species (*Salmonella* ser. Heidelberg and *Salmonella* ser. Minnesota) increased markedly from approximately 60% of isolates in 2014 to >85% in 2017 (Fig. 3). Conversely, there was a notable decrease in the rate of resistance to chloramphenicol in the second sampling period. None of the isolates was resistant to azithromycin or meropenem. Overall, in 2014, 17.8% (26/146) were susceptible to all antimicrobials tested compared with only 6.1% (10/163) in 2017. Multidrug resistance (MDR) was found in 50.7% (74/146) of the isolates from 2014, and this increased to 77.3% (126/163) in 2017 (*P* < 0.05). The highest MDR rates (3–6 antimicrobial classes) were evident for *Salmonella* ser. Heidelberg and *Salmonella* ser. Minnesota. Among the former serotype, MDR rates increased from 78.2% (43/55) in 2014 to 93.3% (83/89) in 2017, and for the latter from 64.7% (11/17) in 2014 and 86.8% (33/38) in 2017.

**Table 2.** Prevalence of antimicrobial-resistant among *Salmonella enterica* from poultry meat in Brazil

ATB	2014 (*n* = 146)		2017 (*n* = 163)		*P* value
	% (no. of isolates)	CI (%)	% (no. of isolates)	CI (%)	
AMP	56.2 (82)	48.0–64.1	76.7 (125)	69.7–82.7	<0.05
AZM	0.0 (0)	0.0–2.0	0.0 (0)	0.0–1.8	–
CAZ	50.0 (73)	41.9–58.1	76.1 (124)	69.1–82.2	<0.05
CHL	6.9 (10)	3.5–11.9	0.6 (1)	0.0–3.0	<0.05
CIP	56.9 (83)	48.7–64.7	89.0 (145)	83.4–93.1	<0.05
CST	3.4 (5)	1.3–7.4	0.6 (1)	0.0–3.0	0.074
CTX	52.1 (76)	44.0–60.1	76.1 (124)	69.1–82.2	<0.05
GEN	6.2 (9)	3.1–11.0	6.1 (10)	3.2–10.7	0.991
MEM	0.0 (0)	0.0–2.0	0.0 (0)	0.0–1.8	–
NAL	57.5 (84)	49.4–65.4	86.5 (141)	80.6–91.1	<0.05
SXT	7.5 (11)	4.0–12.7	4.3 (7)	1.9–8.3	0.225
TET	60.3 (88)	52.2–68.0	82.8 (135)	76.5–88.0	<0.05
TGC	4.8 (7)	2.1–9.3	4.3 (7)	1.9–8.3	0.833

CI, confidence interval; AMP, ampicillin; AZM, azithromycin; CAZ, ceftazidime; CHL, chloramphenicol; CIP, ciprofloxacin; CST, colistin; CTX, cefotaxime; GEN, gentamicin; MEM, meropenem; NAL, nalidixic acid; SXT, sulfamethoxazole/trimethoprim; TET, tetracycline; TGC, tigecycline.

Of note, the resistance profile NAL-AMP-CTX-CAZ-CIP-TET was the most prevalent in both periods, and accounted for 26.0% (38/146) in 2014, and 63.2% (103/163) in 2017. In the earlier sampling period, the great majority 89.5% (34/38) of isolates with this resistance profile were of the serotype Heidelberg, while in 2017, there was a marginal decrease in the prevalence of the latter to 70.9% (73/103) while 25.2% (26/103) were *Salmonella* ser. Minnesota. Combined resistance to ciprofloxacin and cefotaxime was detected in 37.0% (54/146) of the isolates in 2014, and 72.4% (118/163) in 2017; this was also mainly associated with the Heidelberg and Minnesota serotypes.

## Discussion

This survey evaluated non-typhoidal *Salmonella* isolates from the main states involved in Brazilian poultry production, which together account for more than 95% of chicken meat production in the country [8]. Several different *S. enterica* serotypes were identified by automated ribotyping, which has been reported to give data consistent with conventional serotyping [12] and has been used routinely by MAPA laboratories to determine *Salmonella* serovars since 2007. The two most frequent serotypes, among 34 different types (Table S1), identified in both sampling periods (2014 and 2017), were *Salmonella* ser. Heidelberg and *Salmonella* ser. Minnesota. Earlier studies from Brazil also identified *Salmonella* ser. Heidelberg from poultry carcasses and products in rates varying from 0.8% to 5% in isolates from 2007 to 2011 [17]. More recently, this serovar was found to be the most frequent (29.1%) by Fitch *et al*. [18] in poultry meat, being found in three states in Brazil. This increasing trend is confirmed by the current study where it accounted for over one-third and one-half of all isolates, respectively, in the two sampling periods, and from an increased number of states. In the earlier period, isolates of this serovar were restricted to the Southern and Southeast regions of Brazil but by 2017 it had spread through the Southeast and to Midwest regions (Fig. 2).

Our results indicated that *Salmonella* ser. Minnesota is the second most common serotype in poultry carcasses in Brazil, in both 2014 and 2017, accounting for 11.6% (17/146) and 23.3% (38/163) of isolates, respectively. This serotype had already been reported as one of the five most prevalent in poultry carcasses from Brazil between 2007 and 2011, in rates varying from 9.1% to 40.24% [17–19]. Despite the similar prevalence to previous reports, this survey shows clear evidence of the spread of this serotype among the states producing broiler chickens in Brazil, as in 2014, it was identified in six states (SC, PR, MG, GO, MS, MT), while in 2017, it was also identified in ES, PA and SP, encompassing all regions related to chicken meat production in Brazil (Fig. 2).

*Salmonella* ser. Heidelberg is infrequently reported in human and animal sources from European countries [20]. However, it is the second most prevalent serotype in retail poultry meat in Colombia [21]. In the USA and Canada, it is among the top five serotypes from poultry and is frequently associated with invasive human infections [22, 23]. Conversely, *Salmonella* ser. Minnesota has not been significantly associated with human infections and is rarely identified in animals in countries other than Brazil [24]. The shift in the predominant *S*. *enterica* serotypes that occurred in recent years in Brazil is probably associated with the implementation of the control of *Salmonella* ser. Enteritidis and *Salmonella* ser. Typhimurium in poultry farms by the Brazilian Ministry of Agriculture in 2003 [25]. The decline in *Salmonella* ser. Enteritidis, which was the most prevalent serovar in poultry isolates from Brazil until then, would have allowed the occupation of this ecological niche by other serovars, such as *Salmonella* ser. Heidelberg and *Salmonella* ser. Minnesota. A similar phenomenon has occurred in the USA, where *Salmonella* ser. Heidelberg and *Salmonella* ser. Kentucky supplanted *Salmonella* ser. Enteritidis as the predominant serotypes in poultry [17, 22].

*Salmonella enterica* isolates in this study displayed high and increasing rates of resistance, as over 80% were resistant to at least one antibiotic in 2014, and this rose to 94% in 2017. This change was likely due to the increase of MDR strains of *Salmonella* ser. Heidelberg and *Salmonella* ser. Minnesota (Fig. 3).

In 2014, the highest rate of resistance was to tetracycline (60.3%) which increased to 82.8% in 2017 (Table 2). Although tetracyclines have been prohibited for use as zootechnical additives since 1998 in Brazil [26], this class of antimicrobial was one of the first used in animal production and remains approved for therapeutic purposes. Voss-Rech *et al*. [19] also found high levels of resistance to tetracycline (52.4%) in broiler farms in Brazil. Similar results were reported in the USA (53.2% in poultry meat between 2008 and 2017) [27], Canada (44% in broiler chicken between 2013 and 2018) [28] and in countries of the European Union (46.1% in meat from broiler in 2016) [29].

Fluoroquinolones are critically important antimicrobials for human medicine [30] and are the drug of choice to treat invasive salmonellosis in adults [31]. Our results indicated high rates of resistance to nalidixic acid and ciprofloxacin among *Salmonella* isolates from poultry meat. Similar results were also recorded for *Salmonella* in broiler meat from the European Union, with rates in 2016 of 61.5% of resistance for nalidixic acid and 64.7% for ciprofloxacin [29]. Similarly, Colombia reported resistance rates among *Salmonella* of 66.0% for nalidixic acid and 41.2% for ciprofloxacin [21], while in China 99.5% of isolates from broiler chickens were resistant to nalidixic acid, and 48.7% to ciprofloxacin [32]. Voss-Rech *et al*. [33], in a meta-analysis study of the temporal evolution of AMR of non-typhoidal *Salmonella* from humans and poultry in Brazil, had already reported significantly increased levels of resistance to nalidixic acid in humans and poultry isolates, which may be an indicator of emerging resistance to fluoroquinolones in general [34]. On the other hand, fluoroquinolone resistance has been consistently low in *Salmonella* isolates from broiler chicken in Canada [28, 35] and in conventionally produced poultry meat in the USA [27], where these agents are not approved for use in poultry [36, 37]. However, it is important to highlight that the European breakpoint for ciprofloxacin resistance (MIC >0.06 mg/l) is significantly more conservative than that in the USA (MIC >0.5 mg/l). Considering the MIC distribution (data not shown), 56.8% of our isolates from 2014 would be classified as having intermediate susceptibility to ciprofloxacin and none as resistant, according to Clinical and Laboratory Standards Institute (CLSI) breakpoints. In 2017, only two isolates would be considered as resistant, but the great majority would be classed as the intermediate phenotype.

Resistance to extended spectrum cephalosporins is also a serious concern, since these are the antibiotics of choice for treating invasive salmonellosis in children [31]. Our results indicated high and increasing rates of resistance to the third-generation agents, cefotaxime and ceftazidime (Table 2). In Brazil, ceftiofur (a third-generation cephalosporin used exclusively for veterinary medicine) is used to combat early mortality due to infection in chicks, a practice which may promote the selection of resistant strains [17]. Rates of resistance to ceftiofur and ceftriaxone of *Salmonella* ser. Heidelberg obtained from retail chicken carcasses were reported as 43.8% and 75.0%, respectively [38]. In the USA, resistance to ceftriaxone was recently detected in *Salmonella* after the approval of ceftiofur for use in livestock and poultry. This led to the limitation of the use of cephalosporins in food production in the USA and in several other countries [39]. Notably, in Canada, resistance to third-generation cephalosporins, which was mainly associated with the serovar Heidelberg in retail chicken meat, decreased from 21.0% in 2014 to 6.6% in 2016, in *Salmonella* isolates from chicken meat, after the industry banned the use of ceftiofur in broiler chickens in mid-2014 [34].

Combined resistance to fluoroquinolones and third-generation cephalosporins also deserves special attention, owing to the restriction of treatment options for human salmonellosis with combined resistance. In our study, 37.0% of the isolates from samples in 2014 were resistant to both ciprofloxacin and cefotaxime rising to 72.4% in 2017. This resistance phenotype was mainly associated with *Salmonella* ser. Heidelberg, which accounted for 72.2% of the isolates in 2014, and 68.6% in 2017. However, 12 different serotypes also showed this combined resistance profile, including *Salmonella* ser. Minnesota and *Salmonella* ser. Typhimurium. It is noteworthy that among countries of the European Union, this pattern of resistance is very rare, and was previously found only in two *Salmonella* spp. isolates from broiler meat in Belgium, in 2016 [29].

Resistance to polymyxins has regained importance in recent years due to the increasing number of infections caused by carbapenemase-producing *Enterobacteriaceae*, thereby limiting treatment options, and the description of transmissible colistin resistance mediated by the *mcr*−1 gene [40]. Colistin has been used for decades in veterinary medicine and in agricultural production, including as a growth promoter [41]. In Brazil, colistin was prohibited as a zootechnical additive in November 2016 [42], and we found low rates of resistance to this agent in both test periods (Table 2). However, the *mcr-1* gene has been documented in *Salmonella* isolates from pork [43] and poultry meat [44, 45] in Brazil, highlighting the importance of monitoring resistance to colistin in the food chain. In Europe, resistance to colistin was reported by five countries in *Salmonella* isolates from broiler meat in 2016, in rates varying from 1.2% to 16.7% [29]. In the USA, colistin is not marketed or available for use in food-producing animals, and the national surveillance programme (NARMS) do not routinely determine susceptibility to the agent [39].

Regarding other clinically important antibiotics, our study showed very high rates of resistance to ampicillin in both periods (Table 2), which is one of the oldest antibiotics used in veterinary medicine [33]. Similarly, high ampicillin-resistant rates were recorded in *Salmonella* isolates from chicken carcasses in Brazil (38.0%) [38], China (87.8%) [32], Turkey (85.2%) [46] and Mexico (82.9%) [47]. On the other hand, Canada reported declining trends in resistance to ampicillin from 2011 (31.6%) to 2016 (7.1%) [34].

We found no resistance to azithromycin or meropenem among the study isolates. Similar findings for meropenem were reported by countries of the European Union, and azithromycin resistance was minimal [29]. Likewise, our survey identified only low levels of resistance to gentamicin, trimethoprim/sulfamethoxazole and tigecycline, with no trends of increase, while resistance to chloramphenicol decreased significantly over the assessed periods (Table 2). Data from Canada and the USA on *Salmonella* isolates from poultry showed similarly low rates of resistance for these antibiotics [35, 48], except for tigecycline which is not tested by the US authorities for *Salmonella* isolates. In Europe, reported tigecycline resistance rates in 2016 varied from 1.2% to 16.7% from broiler meat [29].

In conclusion, our data provide important baseline information on the serotype distribution and AMR of non-typhoidal *Salmonella* isolates from poultry meat in Brazil. Such data should prove of value for the development and implementation of an Integrated Surveillance Program on Antimicrobial Resistance in Foodborne Pathogens in Brazil. *Salmonella* ser. Heidelberg and *Salmonella* ser. Minnesota were clearly the most frequent serotypes in the two survey periods. High and increasing rates of resistance were recorded for nalidixic acid, ampicillin, cefotaxime, ceftazidime, ciprofloxacin and tetracycline. These results stress the importance of continuous monitoring of AMR in the poultry food chain and the need to expand this surveillance to other food production animals.

## Data Availability

The authors confirm that the data supporting the findings of this study are available within the article and its Supplementary materials.

## Supplementary material

For supplementary material accompanying this paper visit http://dx.doi.org/10.1017/S0950268821002156. click here to view supplementary material
