# Supplementary material for: Antimicrobial resistance of Salmonella from poultry meat in Brazil: results of a nationwide survey
Source: Epidemiol Infect. 2021 Oct 8;149:e228. doi: 10.1017/S0950268821002156 (PMC8569832; doi:10.1017/S0950268821002156)
Supplement: Supplementary file 1 [file S0950268821002156sup001.docx]

***Epidemiology and Infection***

**Antimicrobial resistance of *Salmonella* from poultry meat in Brazil: results of a nationwide survey**

Renata Batista Rau, Aldemir Reginato Ribeiro, Amaury dos Santos, Afonso Luís Barth

**Supplementary material**

Supplementary Table S1: Distribution of *Salmonella* serovars in poultry meat from 2014 and 2017 in Brazil.

| Serotype | % (no. of isolates) | |
| --- | --- | --- |
|  | 2014 (n=146) | 2017 (n=163) |
| Heidelberg | 37.7 (55) | 54.6 (89) |
| Minnesota | 11.6 (17) | 23.3 (38) |
| Schwarzengrund | 6.8 (10) | 1.8 (3) |
| Infantis | 6.2 (9) | 0.6 (1) |
| Saintpaul | 4.1 (6) | 4.9 (8) |
| Molade | 3.4 (5) | 0.0 (0) |
| Anatum | 2.7 (4) | 0.0 (0) |
| Litchfield | 2.7 (4) | 0.0 (0) |
| Brackenridge | 2.1 (3) | 0.0 (0) |
| Javiana | 2.1 (3) | 0.6 (1) |
| Muenchen | 2.1 (3) | 0.0 (0) |
| Agona | 1.4 (2) | 0.6 (1) |
| Manhattan | 1.4 (2) | 0.0 (0) |
| Mbandaka | 1.4 (2) | 1.8 (3) |
| Montevideo | 1.4 (2) | 0.0 (0) |
| Poona | 1.4 (2) | 1.3 (2) |
| Sandiego | 1.4 (2) | 0.0 (0) |
| Senftenberg | 1.4 (2) | 0.6 (1) |
| Typhimurium | 1.4 (2) | 0.0 (0) |
| Enteritidis | 0.7 (1) | 1.3 (2) |
| Alachua | 0.0 (0) | 0.6 (1) |
| Albany | 0.0 (0) | 0.6 (1) |
| Bareilly | 0.0 (0) | 0.6 (1) |
| Derby | 0.7 (1) | 0.0 (0) |
| Give | 0.7 (1) | 0.6 (1) |
| Itami | 0.7 (1) | 0.0 (0) |
| Livingstone | 0.0 (0) | 0.6 (1) |
| Newport | 0.7 (1) | 0.0 (0) |
| Ohio | 0.7 (1) | 0.0 (0) |
| Paratyphi | 0.0 (0) | 0.6 (1) |
| Rissen | 0.7 (1) | 0.6 (1) |
| Shangani | 0.7 (1) | 0.0 (0) |
| Typhimurium/ 4,[5],12:i:- | 0.7 (1) | 0.0 (0) |
| Worthington | 0.7 (1) | 0.0 (0) |
| Unidentified | 0.7 (1) | 4.3 (7) |
